# Methodologies Used to Study the Feasibility, Usability, Efficacy, and Effectiveness of Social Robots For Elderly Adults: Scoping Review

**DOI:** 10.2196/37434

**Published:** 2022-08-01

**Authors:** Aysan Mahmoudi Asl, Mauricio Molinari Ulate, Manuel Franco Martin, Henriëtte van der Roest

**Affiliations:** 1 Department of Research and Development Iberian Institute of Research in Psycho-Sciences INTRAS Foundation Zamora Spain; 2 Psycho-Sciences Research Group Salamanca Biomedical Research Institute Salamanca University Salamanca Spain; 3 Psychiatry and Mental Health Service Assistance Complex of Zamora Zamora Spain; 4 Department on Aging Netherlands Institute of Mental Health and Addiction Trimbos Insititute Utrecht Netherlands

**Keywords:** aged, dementia, social robots, pet-bots, community settings, long-term care, methods, scoping review

## Abstract

**Background:**

New research fields to design social robots for older people are emerging. By providing support with communication and social interaction, these robots aim to increase quality of life. Because of the decline in functioning due to cognitive impairment in older people, social robots are regarded as promising, especially for people with dementia. Although study outcomes are hopeful, the quality of studies on the effectiveness of social robots for the elderly is still low due to many methodological limitations.

**Objective:**

We aimed to review the methodologies used thus far in studies evaluating the feasibility, usability, efficacy, and effectiveness of social robots in clinical and social settings for elderly people, including persons with dementia.

**Methods:**

Dedicated search strings were developed. Searches in MEDLINE (PubMed), Web of Science, PsycInfo, and CINAHL were performed on August 13, 2020.

**Results:**

In the 33 included papers, 23 different social robots were investigated for their feasibility, usability, efficacy, and effectiveness. A total of 8 (24.2%) studies included elderly persons in the community, 9 (27.3%) included long-term care facility residents, and 16 (48.5%) included people with dementia. Most of the studies had a single aim, of which 7 (21.2%) focused on efficacy and 7 (21.2%) focused on effectiveness. Moreover, forms of randomized controlled trials were the most applied designs. Feasibility and usability were often studied together in mixed methods or experimental designs and were most often studied in individual interventions. Feasibility was often assessed with the Unified Theory of the Acceptance and Use of Technology model. Efficacy and effectiveness studies used a range of psychosocial and cognitive outcome measures. However, the included studies failed to find significant improvements in quality of life, depression, and cognition.

**Conclusions:**

This study identified several shortcomings in methodologies used to evaluate social robots, resulting in ambivalent study findings. To improve the quality of these types of studies, efficacy/effectiveness studies will benefit from appropriate randomized controlled trial designs with large sample sizes and individual intervention sessions. Experimental designs might work best for feasibility and usability studies. For each of the 3 goals (efficacy/effectiveness, feasibility, and usability) we also recommend a mixed method of data collection. Multiple interaction sessions running for at least 1 month might aid researchers in drawing significant results and prove the real long-term impact of social robots.

## Introduction

In the next few decades, we expect the global population to age due to a combination of high life expectancy, low birth rates, and the baby boomer generation entering their senior years. By 2030, 33% of the population in Western Europe will be over 60 years of age [1]. Dementia is one of the most common neurodegenerative diseases that affects 50 million older people around the world, and it is projected to reach 155 million in 2050 [2].

Dementia is characterized by deterioration in memory, cognition, behavior, and ability to perform everyday activities [3]. It is estimated that approximately one-third of people with dementia live alone [4]. They experience unmet needs because of living alone and are at a higher risk of faster deterioration. In addition, people with dementia who live alone are considered at a higher risk of medication use problems, falls and injuries, inadequate self-care, trouble with activities of daily living, and reduced social networks [5-8].

In the past decades, technological advances coincided with the great health challenge of aging societies [9]. New research fields in assistive technology are dedicated to designing social robots for older adults with or without cognitive impairment to promote their quality of life (QoL) through communication and social interactions [10]. Social robots are intended to provide and facilitate social contact, psychosocial and cognitive stimulation, and have the potential to support elderly people to maintain their autonomy and independence and enhance their well-being [11].

Socially assistive robots (SARs) can be grouped into 2 main categories based on their feature and function: (1) service robots, and (2) companion robots [12]. The main task of these robots is to establish any form of interaction and communication. This function can be performed by SARs in multiple manners, such as through touch sensors, cameras, (robotic) body movements, tablet interfaces, and sound and speech systems. Within the subgroup of the companion robots, humanoid robots like Pepper and Nao provide users with advanced applications that provide leisure activities (music, photos, and games), cognitive and physical stimulation activities, and assistance with mental or physical tasks. Pet robots, such as PARO, AIBO, and NeCoro as substitutes for pets and companion animals are intended to provide emotional and physiological stimulation, have calming effects, and lead to mood improvements [13].

For the successful implementation and large-scale uptake of social robots or any other psychosocial intervention, their feasibility, usability, and cost-effectiveness should be perceived as good by the end users (people with dementia and healthy older adults), clinicians, and other stakeholders (eg, health care insurers and policy makers). The Monitoring and Evaluating Digital Health Interventions framework recommends evaluating 4 factors to integrate and implement a digital health intervention: (1) feasibility, to assesses whether the digital health system works as intended in a given context; (2) usability, to assess whether the digital health system can be used as intended by users; (3) efficacy, to assess whether the digital health intervention can achieve the intended results in a research (controlled) setting, and (4) effectiveness, to assess whether the digital health intervention can achieve the intended results in a nonresearch (uncontrolled) setting [14].

Despite the rising interest in social robots after the COVID-19 pandemic, there is limited evidence on the effectiveness of social robots in elderly care. The methodological quality of studies on the effectiveness of social robots in elderly adults is still low, and inappropriate study designs, samples, form, duration of interventions, and data collection methods have affected the strength of study outcomes [12].

Currently, there is no state-of-the-art proof of concept for study designs to evaluate the use of social robots for elderly people. Since the degenerative nature of dementia can cause methodological challenges, specific attention should be paid to studies that include people with dementia. To determine what the appropriate research methods are to study feasibility, usability, efficacy, and effectiveness, this article aims to review the methodologies used thus far in studies with social robots in clinical and social settings with elderly people to pave the way for future researchers in this field.

## Methods

### Protocol Registration

The protocol of this scoping review was registered in Open Science Framework (OSF) [15].

### Search Strategy

Searches were conducted on August 13, 2020, in MEDLINE (PubMed), Web of Science, PsycInfo, and CINAHL databases. No time window was applied. Three search strings covering the topics “social robots,” “community setting,” and “elderly people” were constructed. For each database, reverent subject headings were adapted. For MEDLINE, we used the following strings and keywords: ((robotics[MeSH Terms] OR robot*)) AND ((humanoid OR companion OR social* OR “socially assistive” OR interact* OR android)), (((((((aging[MeSH Terms]) OR (aged[MeSH Terms])) OR (elderly[MeSH Terms])) OR (vulnerable population[MeSH Terms])) OR (senior)) OR (ageing)) OR (geriatric)) OR (old*), and (((((community health service[MeSH Terms]) OR (social support[MeSH Terms])) OR (residential facilities[MeSH Terms])) OR (independent living[MeSH Terms])) OR (social support[MeSH Major Topic])) OR (“community dwelling” OR “home dwelling” OR “care home” OR “in-home” OR “at home” OR “home-based” OR “home setting” OR “nursing home” OR home).

### Selection Criteria

Publications potentially eligible for inclusion had to study a social robot that was physically embedded in an experimental or clinical study in people aged 65 or above. Studies were excluded if they were (1) conducted in an acute care setting; (2) conference abstracts, case studies, dissertations, books, or review papers; (3) published in a language other than English or Spanish; (4) solely reporting a robot design, development process, or theoretical models (5) stakeholder opinions on robots without any interaction; (6) involved in the implementation of new hardware or software or an assessment tool on a robot (such as assessing a fall detection sensor); and (7) involving telepresence robots with only video call features.

### Selection Procedure

After the removal of duplicates, 2 reviewers (authors AM and MM) independently applied the inclusion and exclusion criteria in 3 steps, starting with screening titles, abstracts, and then full texts. The selections were compared, and in case of disagreement, discussed by the 2 reviewers. In cases where no consensus could be reached, a third reviewer was consulted (author HR).

### Data Extraction

The literature was mapped according to the following areas of interest: author and country, robot name, the aim of the robot, aim of the study, type of outcome measure, study design, study sample, study setting, methodology of data collection, interaction scenario, relevant outcome measures, measurement instruments, results, and reported limitations of the study. Information was synthesized descriptively, and findings were narratively summarized according to the areas of interest.

The quality of the included articles was appraised independently by 2 authors (AM and MM), through the quality assessment of digital health interventions within the Monitoring and Evaluating Digital Health Interventions framework established by the World Health Organization (WHO) [14]. The tool includes a list of methodological study criteria comprising (1) 23 essential criteria for all types of studies and (2) essential criteria for qualitative and quantitative studies (3 criteria each). The extent to which criteria were met by studies was rated by 3 independent researchers on a 3-point scale (0= poor, 1= fair, 2= good)*.* We calculated the percentage of agreement between the ratings (Multimedia Appendix 1). We also applied this framework to categorize the studies according to their aims in 4 categories: (1) feasibility, (2) usability, (3) efficacy, and (4) effectiveness.

## Results

### General Findings

**Figure 1:**
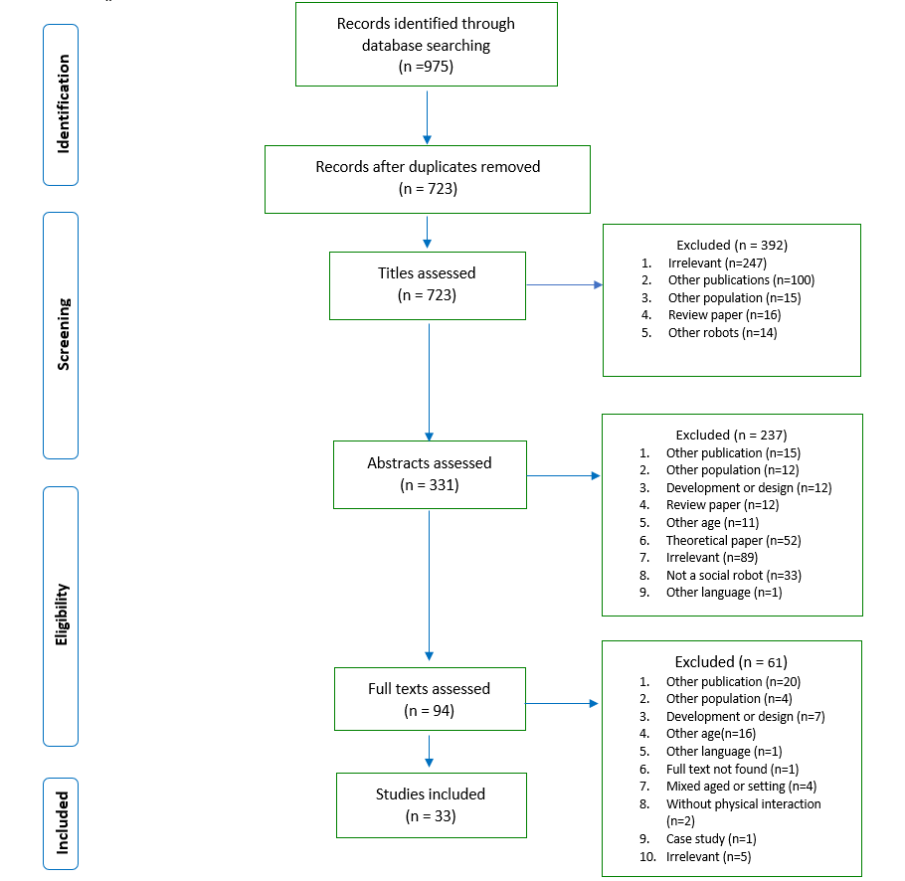
PRISMA (Preferred Reporting Items for Systematic Reviews and Meta-Analyses) flow diagram for literature search.

The search resulted in a total of 723 individual publications. After the screening process, 33 articles met the selection criteria (Figure 1). In 33 papers [11,16-45], 23 different social robots were evaluated among elderly adults and people with dementia in 13 different countries. Moreover, 19 studies specifically evaluated either feasibility, usability, efficacy, or effectiveness and were considered as single aim studies. The remaining studies (n=14) had multiple aims, evaluating 2 or 3 of the aforementioned study aims. Overall, feasibility was studied in 17 (51.5%) studies, usability in 13 (39.3%), effectiveness in 12 (36.3%), and efficacy in 10 (30.3%).

The quality appraisal identified that primary and secondary outcomes were clearly defined in all studies. Additionally, the methods of data collection were described well, but the eligibility of the participants was not reported in 12 (36.4%) papers. Moreover, 12 out of 33 (36.4%) papers did not present a clear description of the study design. Multimedia Appendices 2 and 3 show a summary of the characteristics, methodologies, and outcomes of the included studies.

### Robots

Of the 23 different social robots, PARO (n=10, 30.3%), Nao (n=5, 15.1%), AIBO (n=2, 6%), and Hobbit (n=4, 12.1%) were the most often investigated.

### Participants and Settings

Of the 33 identified studies, 16 (48.4%) focused on people with dementia [11,18,23,24,27,29-35,38,39,43,44], 9 (27.3%) were performed in samples of residents of long-term care facilities whose cognitive status was not mentioned [17,20,22,26,28,36,40,41,45], and the remaining 8 (24.2%) focused on elderly people living in the community whose cognitive status was also not clearly revealed [16,19,22,25,37,42,46,47]. Moreover, 4 (12.1%) studies additionally recruited care staff [22,24,26,38]. The age range of older adults was 65-98 years. Of the included studies, 3 (9.1%) did not report the number of participants [17,20,26]. The sample sizes used in the studies ranged from 5 to 139.

The social robots were studied in long-term facilities (n=17, 51.5%), private households (n=8, 24.2%), and laboratory settings (n=4, 12.1%). Additional settings were based in a care organization (n=1, 3%), a daycare center for dementia (n=1, 3%), and a health service facility (n=1, 3%). Four (12.1%) studies investigated the robots in 2 different settings [22,33,43,46].

### Study Aims, Designs, and Outcome Measures

#### Single Aim Studies

Of the included studies, 3 (9.1%) focused solely on feasibility, using quasi-experimental designs [22,27,46], and 1 (3%) explicitly focused on usability in a private home setting [37]. Additionally, 7 (21.2%) studies aimed at studying efficacy [31-35,38,44], of which 2 (28.5%) applied a form of randomized controlled trial (RCT) design, 1 (14.3%) randomized crossover design, 1 (14.3%) pretest-posttest design, and the other 3 (42.8%) a form of quasi-experimental design. The effectiveness of the robots was explicitly studied in 8 (24.2%) articles [17,20,24,36,40,41,43,45] using randomized designs, with 1 (12.5%) RCT, 2 (25%) blocked RCTs, 2 (25%) quasi-experimental designs, 1 (12.5%) pretest-posttest design, 1 (12.5%) cross-sectional, and 1 (12.5%) qualitative study. The impact of robots was evaluated on QoL (n=6, 18.2%), mood and depression (n=6, 18.2%), behavioral (n=6, 18.2%) and neuropsychiatric symptoms (n=2, 6.1%), emotions and affect (n=5, 15.2%), cognition (n=4, 12,1%), engagement (n=8, 24.2%), participation and social interaction (n=8, 24.2%), care burden (n=1, 3%), loneliness (n=1, 3%), and physiological indicators (n=3, 9.1%). The sample size for the 22 studies with effectiveness/efficacy aims ranged from 11 to 139 participants, and 7 (31.8%) of these studies included samples of over 40 participants.

#### Multiple Aim Studies

A total of 14 (42.4%) studies had multiple aims. Of these, 7 (50%) focused on feasibility and usability [11,16,19,22,25,28,44], of which 3 (42.9%) applied a mixed methods design, and the remaining 4 (57.1%) applied either an experimental design or a field trial. Meanwhile, 3 (9.1%) focused on feasibility, usability, and effectiveness, and all applied a mixed methods design [26,29,39]. Additionally, 1 (3%) study investigated feasibility and efficacy and applied an experimental design [30], 1 (3%) focused on feasibility, usability, and efficacy using a pretest-posttest design [18], and 1 (3%) assessed the feasibility and effectiveness of the robot, applying a nonrandomized controlled trial design [22].

### Study Aims and Settings

Only 5 (27.8%) of the 18 studies aiming to evaluate feasibility and/or usability were performed in nursing home settings; 5 (27.8%) were performed in laboratory settings, and the remaining 8 (44.4%) were performed in private households. In 7 (38.9%) of the 18 studies, people with dementia and those with cognitive impairment were included. In the remaining 11 (61.1%) studies, the cognitive status of the participants was not clearly indicated.

Of the 22 (66.7%) studies that focused on efficacy or effectiveness, all but 4 (81.8%) [29,33,39,47] were performed in long-term care settings. These 4 (18.2%) were performed in private households and a daycare facility. Of these studies, 13 (59.1%) included cognitively impaired samples, only 1 (4.5%) study included community-dwelling elderly persons without disclosing their cognitive status, and the remaining 8 (36.4%) included long-term care residents.

### Study Interventions

Interaction between study participants and social robots was mostly investigated during individual sessions (n=18, 54.5%). In 12 (36.4%) studies, interactions were studied in group sessions. Only 3 (9.1%) studies applied both individual and group interactions [11,20,33], while 1 (3%) demonstrated the task performance of the robot without any close interaction with study participants [19]. Feasibility and/or usability (n=11, 33.3%) were mostly studied in individual settings (n = 8, 72.7%); 1 (9.1%) study was performed in a group, and 1 (9.1%) was applied to both individual and group settings. Individual (n=7, 21.2%) and group settings (n=6, 18.2%) were used most often to study efficacy and effectiveness (n=15, 45.5%); 2 (6.1%) studies applied the intervention individually and in a group. In studies with multiple aims, 4 (28.6%) individual and 2 (14.3%) group setting interventions were found. In 2 (6.1%) studies, social robots were available in the residents’ lounge in nursing homes, and participants were free to interact with the social robots during scheduled time slots [22,45]. In 8 (24.2%) studies, the robots were installed in participants’ private homes for a duration of 5 days to 3 months [16,27,29,33,37,39,44,47].

A total of 9 (27.3%) studies executed 1 or 2 interactive sessions [11,19,22-24,28,30,32,34], of which 6 (66.7%) investigated the usability and feasibility of the robot, 1 (11.1%) investigated effectiveness, and 2 (22.2%) investigated efficacy. Most of the studies conducted more than 2 interactive sessions: 5 (15.2%) studied feasibility and/or usability, 12 (36.4%) studied efficacy or effectiveness, and 8 (24.2%) were multiple aim studies. The interactive sessions ran from 10 to 90 minutes a day for a maximum of 4 months.

### Data Collection

**Figure 2:**
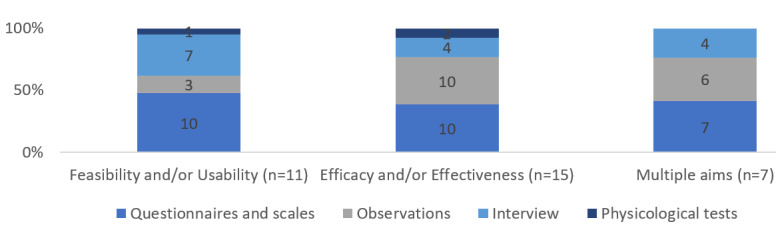
Used methods of data collection for single and multiple aim studies.

**Figure 3:**
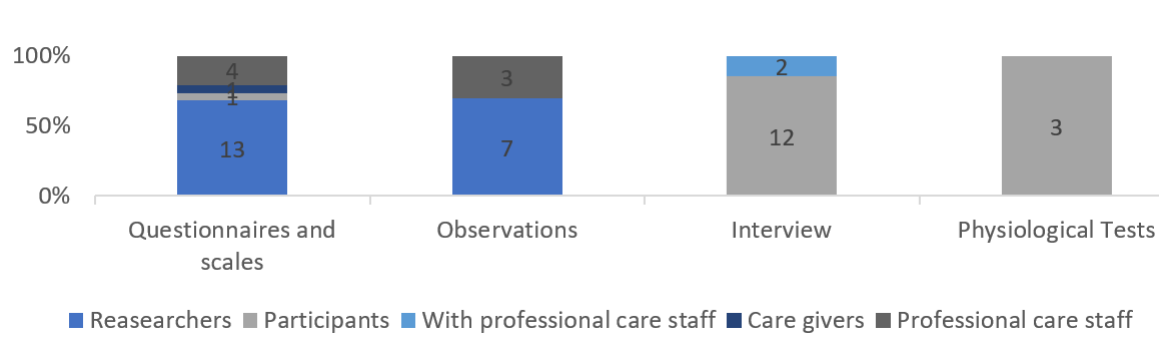
Responsible administrator of data for identified methods of data collection.

We identified 4 methods of data collection: (1) questionnaires (n=26, 78.8%), (2) observations (physical and videotape) (n=19, 57.6%), (3) interviews (n=13, 39.4%), and physiological measurements (n=3, 9.1%). Figures 2 and 3 show the data collection methods and the responsible administrator of data for the identified data collection methods, respectively.

### Measurement Instruments

Outcomes regarding feasibility were assessed with the Unified Theory of the Acceptance and Use of Technology (UTAUT) model adapted by Heerink [11,19,23,29,30,46,48], the Robot User Acceptance Scale, the Robot Attitude Scale, the Mind Perception Scale [21,47], and the Negative Attitudes Toward Robots Scale [16,37]. Of the included studies, 5 (15.2%) utilized questionnaires regarding robot functions and acceptance that were specifically developed for the study [19,22,23,25,46].

Studies exploring usability applied the System Usability Scale [22,25,28], a modification of the Usefulness, Satisfaction, and Ease of Use [26] scale, and the Technology Usage Inventory [39]. Two (6.1%) qualitative studies performed conversation and video analysis [27,28] to extract statements on acceptability and usability.

Efficacy and effectiveness outcomes were evaluated by a wide range of neuropsychosocial measurement instruments: (1) mood: Geriatric Depression Screening [49], the Cornell Scale For Depression in Dementia [50], Apparent Emotion Rating Instrument [51], University of California, Los Angeles (UCLA) Loneliness Scale [52], Observed Emotion Rating Scale [53]; (2) cognition: Montreal Cognitive Assessment [54], Mini-Mental State Examination [55]; (3) QoL: QoL Alzheimer Disease, Dementia QoL Questionnaire [56], QoL in Late-Stage Dementia [57]; (4) behavior: Neuropsychiatric Inventory [58], Gottfries-Bråne-Steen Scale [59], Apathy Evaluation Scale [60], Cohen-Mansfield Agitation Inventory [61], Apathy Inventory [62], Apathy Scale for Institutionalized People With Dementia Nursing Home Version [63]; (5) Participation and Interaction: Activity Participation Scale [40], and Assessments of Communication and Interaction Skills [64].

Among the studies applying questionnaires, 5 (19.2%) indirectly collected data via care staff and informal caregivers, and 13 (50%) directly collected data via the researchers.

### Study Outcomes

Concerning social robots’ feasibility outcomes, almost all studies (n=16, 94.1%) deemed social robots acceptable. Nevertheless, 1 (5.9%) study reported mixed results on acceptability by care staff [22], and 1 (5.9%) did not find any significant results on quantitative measurements for acceptability, but qualitative results were positive [16]. In 3 (17.6%) studies, the perceived agency [21,47] and perceived enjoyment [46] were found to decrease over time.

The reported usability (n=12, 36.4%) was overall positive, except in 2 (16.7%) studies in which the usability was negatively affected by technical issues or lack of robustness of the robots [28,37]. Only 3 (9.1%) studies assessed affordability for Hobbit and Nao, in which the participants did not consider the social robots affordable and were skeptical of buying them [25,28,37].

Most of the findings endorse the use of social robots by older adults. Improvements were mostly found in emotion and mood [20,31,33-35,38,44], engagement [24,29,30], and participation and social interaction [20,31-33,40,45]. Increased job satisfaction of staff [22], self-report pain reduction [38], and improved global psychiatric symptoms [43] were the other positive study outcomes. There were findings of reduced challenging behavior [20,31,34], sense of loneliness [17], and stress levels [45]. However, dementia symptoms like agitation and other problematic symptoms did not improve in 1 (3%) study [33].

Meanwhile, 4 (12.1%) studies did not find a significant impact on QoL [19,21,43,47]. Only 1 (3%) study found a moderate-to-large positive effect on QoL of people with dementia [35]. Social robot interventions also failed to significantly improve depression [18,21,43,47], perceived social support [18], medication adherence [47], and cognition [24]. There were mixed results regarding physiological measures, such as urine tests measuring stress levels and blood pressure [33,45]. No author declared a proven negative effect of social robots on older adults.

### Reported Study Limitations

Of the 33 studies, 7 (21.2%) did not report any study limitations [16,17,23,25,30,42,46]. A wide range of limitations was reported, and the most common barrier considered in 17 (51.5%) studies was the small sample size, which was mostly reported for efficacy and effectiveness studies. In the feasibility and/or usability studies, the limitations were mainly attributed to technical problems or interaction difficulties. The use of unvalidated questionnaires, homogeneity in the sex of the study sample, inadequate observation, and short duration of interventions were reported as other limitations in general.

### Quality Appraisal

The inter-rater agreement for the quality appraisal was 86.1%. Reports of the description of study design, bias, and enrollment procedure were mostly rated as “fair.” In most of the articles, the sampling methods, confounding factors, missing data in quantitative studies, and reflexivity of data interpretation in qualitative studies were poorly reported. Other criteria were mostly rated as “good” (Multimedia Appendix 1). The quality appraisal revealed unclear descriptions or insufficient details in 5 (15.2%) studies, especially those in disciplines other than health research [25,30,42,44,45].

## Discussion

### Principal Findings

The results of this scoping review revealed a variety of applied study methods in studies with social robots concerning study design, sample size, study setting, method of data collection, interaction scenario (the sequence, duration, and setting of the intervention), outcome measures, measurement instruments, study results, and reported limitations. Feasibility and usability were mainly studied on preprototype social robots in laboratory settings. Considering the relatively short history of the use of social robots in psychosocial interventions, it is crucial to determine the main features and functions of the robots to be considered in the design and development phase. Hence, usability, feasibility, and implementation should be strategic research aims. Fully developed robots such as PARO were evaluated in terms of effectiveness in real-world settings. Most of the identified studies aimed to determine the neuropsychosocial impact of social robots on older adults.

For the studies that explicitly fall within a feasibility and/or usability evaluation, researchers applied experimental, mixed method, and field trial designs, mostly applied outside nursing home care settings. This might imply that feasibility and/or usability for persons that are more severely cognitively impaired are currently understudied. Most of the studies verified the acceptability and usability of the robots within single or multiple interactive sessions in individual or group settings, and all these studies reported positive outcomes in varying degrees on the feasibility and/or usability of the social robots. The quantitative and qualitative data were collected mostly through questionnaires and interviews and a few by direct observation. Regarding this point, researchers should consider using the direct observational methodology to capture main factors of the interaction and emotional relationships fostered by robot use. Within the questionnaires and interview questions based on the UTAUT model, some concepts such as trust, anxiety, perceived enjoyment, and social support can change over time [37,46,47]. Therefore, longer use of the robots might reveal these changes and reduce the novelty effect over time [46].

Overall, efficacy and effectiveness studies were conducted on study populations either with cognitive impairment or residing in long-term care facilities. The studies with significant results [17,24,29-31,34-36,45] mostly employed experimental designs including RCTs and quasi-experimental designs with larger sample sizes and longer intervention periods compared to studies showing slight or no improvements. RCTs are likely to be the most appropriate design and a gold standard to confidently demonstrate that a specific intervention has resulted in a change in a process or a health outcome [14]. Biased assessment of outcomes and any confounding effects can be avoidable by large-scale RCTs. However, due to the degenerative character of dementia and personal differences in capacities of people with dementia, difficulties in randomizing subjects often arise [14]. Additionally, when using long study periods, the dropout rate might be high, as participants’ cognitive deterioration can hinder their continued participation in the study. On the other hand, when it is not feasible or ethical to conduct an RCT, a quasi-experimental design may serve best for collecting quantitative data. We also recommend randomized controlled block designs in the case of heterogeneous study samples. When, for instance, including people with dementia in studies with long intervention periods, the dementia levels alter. With a randomized controlled block design, some variables in different blocks can be controlled for, or comparable approaches can be applied within the blocks.

Studies targeting the efficacy and effectiveness of the robots delivered interventions diverged in format, duration, dosage, and location. Two (6.1%) studies [32,38] highlighted a need for individual intervention sessions tailored to users’ preferences and capacities, and the findings of another study confirmed this approach [65]. Additionally, individualized sessions could omit confounding factors by reducing the chance of interactions with the facilitator or other participants [66]. Group interventions may lower the odds of interaction between potential users and the robot, potentially lowering the effect of the intervention, especially when the intervention is delivered in a noisy setting with many participants [18]. The issues of small sample sizes and short interactions were considered major limitations in studies that failed to find significant results, and they are considered the toughest challenges for researchers in this field [66,67]. These limitations are often cojoined with the study setting. In nursing homes, a larger number of residents are often enrolled in a clinical trial, and the robots are not personalized but must be shared by the entire group. Whereas in private homes, studies are conducted with individuals or dyads, which creates better possibilities for personalization of the robot. Overall, the personalization of the intervention and alleviation of loneliness are 2 advantages of home-based clinical trials. However, there are some challenges to these types of studies, such as the need for several robots, implementation difficulty, and personalization, but it is nevertheless a step in the right direction. We observe a paradox, in that new or experimental robots are employed in research with low numbers of participants, whereas commercially available robots are tested on large study samples. Commercialization allows for better testing and evaluation, which is logical from an economical perspective. However, we urge that before robots are marketed, developers should study the feasibility and usability appropriately in the target group, as well as the effectiveness in a substantial study sample with sufficient power. After bringing the robot to the market, producers should continue to invest in studies to improve their product to tailor it optimally to their users. This should be a joined mission of producers and policy makers to improve the sustainability of health care systems.

Apart from the aforementioned limitations of the studies, some weak aspects of the study designs led to failure of the social robots’ impacts. For instance, a mismatch between the studied construct and the main aim of the robot may lead to the poor conclusion that the robot is not efficient. An example of this is the studies on PARO that failed to demonstrate significant results for cognition, as PARO is not developed to stimulate cognitive functioning [31,33,41,43]. Additionally, to capture significant results in constructs such as cognition, QoL, and depression, a long intervention period is necessary because these are constructs that do not change very quickly. In studies with people with dementia, it might also be useful to study stability of these constructs instead of improvement, since it is inherent to the disease that these constructs deteriorate over time. Regarding the broad concept of QoL ranging from physical health to psychological state and social relationships, the application of a suitable QoL measurement instrument that corresponds to the robot’s aim should be taken into account.

### Implications For Efficacy and Effectiveness Studies

Appropriate RCTs with large sample sizes and individual interaction sessions running for longer than 1 month would serve best for such studies to draw relatively robust and reliable results.

### Implications For Feasibility and Usability Studies

The study methods are similar for both aims, so researchers could apply the same design. Experimental designs with mixed methods of data collection are recommended for these studies. Multiple interaction sessions might reveal the changes in feasibility and usability.

### Implication For Studies With Multiple Aims

We recommend performing separate studies for multiple aims since the study designs for each aim are comparable.

We gathered further practical recommendations through which future work may address existing shortcomings (Table 1). Regarding the mixed methods of data collection, studies suggest a combination of qualitative and quantitative methods for data collection, which will enable the researcher to capture different details in users’ responses and address different aspects of the research question. A mixed methods approach was helpful in studies that could not derive positive results from quantitative data but did from qualitative data [16]. Regarding the difficulties of recruiting many users in case of availability of just a few robots, these mixed methods should be mandatory. Even though we did not find any negative results regarding the intervention dosage, there are shreds of evidence of highly intense intervention resulting in negative effects or exhaustion [18]. Hence, the dose response for specific measures remain an open question for future researchers.

**Table 1:** Further implications and recommendations for future studies.

Area of consideration	Type of study	Recommendation
Participant and setting	Efficacy/effectiveness	Gender homogeneity Different levels of dementia Realistic environments
Intervention and data collection	Efficacy/effectiveness	Multiple intervention sessions for longer than 1 month
	Feasibility/usability	Initialization phase before trial
	All types of studies	Well-trained observers and professionals Tailored interventions Include an intervention facilitator apart from an observer Consistent observations Standardized and validated measurement instruments A client-centered approach to intervention design A combination of qualitative and quantitative methods of data collection Observational study when including people with severe dementia
Gap in the existing literature to be filled	Efficacy/effectiveness	Best response-dosage of intervention for particular measure and participant condition Characteristics of subjects who benefit most from the social robots

### Limitations and Strengths

Although the use of social robots is promising to support people with dementia, we did not include dementia specifically in the search strings, since this scoping review focused on elderly people in general. However, we believe that our search captured most of the studies executed in the field of dementia because many of the identified studies included people with dementia in either mixed or specific study samples. However, some relevant studies on elderly people with dementia may be missing in this review, as well as may studies that are only traceable in databases that were not taken into account in this review. The searches were conducted in scientific databases deemed the most viable to retrieve valid and reliable results for this scoping review. The exclusion of studies focusing only on the development phase of social robots can be considered a limitation of this study. Some information on the evaluation of the feasibility and usability executed in the development stage might have been missed. In addition, studies on telepresence robots were excluded due to their relatively simple features. Compared to pet robots and humanoid robots, telepresence robots are limited in interactions with users, which occur merely through a touch screen, making use of visual and audio stimuli but omitting other sensory stimulation. Although mainly used for medical visits, some telepresence robots might support social functioning. Information on studies performed on these robots might have been missed. 

Our study is the first scoping review on the methodologies for studying social robots in elderly people and people with dementia. The existing reviews on this topic mostly focus on design, use, effectiveness, facilitators, and barriers to the implementation of social robots [12,66,67,68-73]. This study might support future researchers to design a research study on social robots in elderly adults and answer some study design queries.

### Conclusions

This review narratively synthesizes information on the methodology of studying social robots in elderly adults and people with dementia. Relevant recommendations were formulated, directed for studies with specific aims that may aid future researchers in developing adequate study designs to evaluate social robots, allowing for more reliable information on study outcomes. Our research leads us to the conclusion that more studies with large sample sizes are needed on the effectiveness of social robots in private households of elderly adults and people with dementia to demonstrate the actual usefulness of social robots on delaying institutionalization by improving QoL, cognition, and social contact, and counteracting loneliness. Most of the identified studies focused on usability, and the robots appeared to be favorably accepted in most cases. It is time to encourage investigations in private homes to supplement existing knowledge about the effectiveness of robots and personalization of their functions. We expect that additional research will corroborate the impact of social robots on loneliness, mood, QoL, and social health in people with dementia and the elderly.

## Appendix

Multimedia Appendix 1 Quality appraisal of the included studies assessed by two independent reviewers.

Multimedia Appendix 2 Characteristics and methodologies of the studies.

Multimedia Appendix 3 Outcomes.

## Abbreviations

ADL: activities of daily living
ITN: innovative training network
LTCF: long-term care facility
OSF: Open Science Framework
QoL: quality of life
RCT: randomized controlled trial
SAR: socially assistive robot
UCLA: University of California, Los Angeles
UTAUT: Unified Theory of the Acceptance and Use of Technology
WHO: World Health Organization
