# Supplementary material for: Methodologies Used to Study the Feasibility, Usability, Efficacy, and Effectiveness of Social Robots For Elderly Adults: Scoping Review
Source: J Med Internet Res. 2022 Aug 1;24(8):e37434. doi: 10.2196/37434 (PMC9379790; doi:10.2196/37434)
Supplement: Multimedia Appendix 1 [file jmir_v24i8e37434_app1.docx]

| **Table 2.** Quality appraisal of the included studies assessed by two independent reviewers | | | | | | | | | | | | | | | | | | | | | | | | | | | | | | |
| --- | --- | --- | --- | --- | --- | --- | --- | --- | --- | --- | --- | --- | --- | --- | --- | --- | --- | --- | --- | --- | --- | --- | --- | --- | --- | --- | --- | --- | --- | --- |
| Study | Introduction | | | Methods | | | | | | | | | | Results | | | | | | | Conflicts | | | Quantitative study | | | Qualitative criteria | | |  |
|  | Rationale | Objectives | Logic model | Study design | Outcomes | Data collection methods | Participant eligibility | Recruitment | Bias | Sampling | Setting and location | Comparator | Data sources | Enrolment | Description of study population | Reporting on outcomes | Summary of evidence | Limitations | Generalizability | Conclusions | Funding | Ethical considerations | Competing interests | Confounding | Statistical methods | Missing data | Analytical methods | Data validation | Reflexivity of account provided | Quality scores |
| Bajones, et al [16] | 2 | 2 | 2 | 1 | 2 | 2 | 2 | 0 | 1 | 1 | 1 | N/A | 2 | 1 | 2 | 2 | 2 | 0 | 0 | 2 | 0 | 0 | 0 | 0 | 1 | 0 | 2 | 2 | 0 | 1.4 |
|  | 2 | 2 | 0 | 2 | 2 | 2 | 2 | 0 | 2 | 0 | 2 | N/A | 2 | 1 | 1 | 2 | 2 | 0 | 0 | 2 | 2 | 2 | 2 | 0 | 1 | 0 | 2 | 2 | 0 |  |
| Banks, et al [17] | 2 | 2 | 2 | 0 | 2 | 2 | 1 | 2 | 1 | 0 | 1 | 2 | 2 | 0 | 0 | 2 | 2 | 0 | 0 | 2 | 0 | 1 | 0 | 0 | 2 | 0 | N/A | N/A | N/A | 1.1 |
|  | 2 | 2 | 2 | 1 | 2 | 2 | 2 | 2 | 0 | 0 | 1 | 2 | 2 | 0 | 0 | 2 | 2 | 0 | 0 | 2 | 0 | 1 | 0 | 0 | 2 | 0 | N/A | N/A | N/A |  |
| Barrett, et al [18] | 2 | 2 | 0 | 1 | 2 | 2 | 2 | 2 | 2 | N/A | 1 | N/A | 2 | 2 | 2 | 2 | 2 | 2 | 2 | 2 | 2 | 2 | 2 | 0 | 2 | 0 | N/A | N/A | N/A | 1.7 |
|  | 2 | 2 | 0 | 2 | 2 | 2 | 2 | 2 | 2 | N/A | 1 | N/A | 2 | 2 | 2 | 2 | 2 | 2 | 2 | 2 | 2 | 2 | 2 | 0 | 2 | 0 | N/A | N/A | N/A |  |
| Beer, et al [19] | 2 | 2 | 2 | 0 | 2 | 2 | 0 | 2 | 2 | 0 | 2 | 2 | 2 | 0 | 2 | 2 | 2 | 2 | 2 | 2 | 2 | 0 | 0 | 0 | 2 | 0 | 2 | 2 | 0 | 1.6 |
|  | 2 | 2 | 2 | 2 | 2 | 2 | 0 | 2 | 2 | 0 | 2 | 2 | 2 | 0 | 2 | 2 | 2 | 2 | 2 | 2 | 2 | 2 | 0 | 0 | 2 | 0 | 2 | 2 | 0 |  |
| Birks, et al [20] | 2 | 2 | 0 | 1 | 2 | 1 | 0 | 0 | 1 | N/A | 2 | N/A | 0 | 0 | 0 | 2 | 2 | 2 | 2 | 2 | 0 | 2 | 2 | N/A | N/A | N/A | 2 | 2 | 0 | 1.2 |
|  | 2 | 2 | 0 | 2 | 2 | 2 | 0 | 0 | 0 | N/A | 2 | N/A | 0 | 0 | 0 | 2 | 2 | 2 | 2 | 2 | 0 | 0 | 2 | N/A | N/A | N/A | 2 | 2 | 0 |  |
| Broadbent, et al [21] | 2 | 2 | 0 | 2 | 2 | 2 | 0 | 2 | 1 | N/A | 2 | 2 | 0 | 2 | 2 | 2 | 2 | 1 | 0 | 2 | 2 | 2 | 0 | 0 | 2 | 0 | N/A | N/A | N/A | 1.4 |
|  | 1 | 2 | 0 | 1 | 2 | 2 | 0 | 2 | 1 | N/A | 2 | 2 | 2 | 2 | 2 | 2 | 2 | 2 | 0 | 2 | 2 | 2 | 0 | 0 | 2 | 0 | N/A | N/A | N/A |  |
| Broadbent, et al [47] | 2 | 2 | 0 | 2 | 2 | 2 | 0 | 2 | 0 | 0 | 2 | 2 | 2 | 2 | 2 | 2 | 1 | 1 | 0 | 1 | 2 | 2 | 0 | 0 | 2 | 0 | 0 | 0 | 0 | 1.2 |
|  | 2 | 2 | 0 | 2 | 2 | 2 | 1 | 2 | 2 | 0 | 2 | 2 | 2 | 2 | 1 | 2 | 1 | 0 | 0 | 2 | 2 | 2 | 0 | 0 | 2 | 0 | 0 | 0 | 0 |  |
| Cavallo, et al [22] | 2 | 2 | 0 | 0 | 2 | 2 | 2 | 2 | 2 | 0 | 1 | 2 | 2 | 2 | 2 | 2 | 2 | 2 | 1 | 2 | 2 | 0 | 2 | 0 | 2 | 0 | N/A | N/A | N/A | 1.6 |
|  | 2 | 2 | 0 | 1 | 2 | 2 | 2 | 2 | 2 | 0 | 1 | 2 | 2 | 2 | 2 | 2 | 2 | 2 | 1 | 2 | 2 | 2 | 2 | 0 | 2 | 0 | N/A | N/A | N/A |  |
| Cavallo, et al [23] | 2 | 2 | 2 | 0 | 2 | 1 | 2 | 2 | 0 | 0 | 1 | N/A | 0 | 0 | 2 | 2 | 2 | 0 | 0 | 2 | 2 | 0 | 0 | 0 | 0 | 0 | N/A | N/A | N/A | 1.1 |
|  | 2 | 2 | 2 | 1 | 2 | 2 | 2 | 2 | 0 | 0 | 1 | N/A | 0 | 0 | 2 | 2 | 2 | 0 | 0 | 2 | 2 | 2 | 0 | 0 | 0 | 0 | N/A | N/A | N/A |  |
| Chu, et al [24] | 2 | 2 | 2 | 2 | 2 | 2 | 0 | 0 | 2 | N/A | 1 | N/A | 2 | 0 | 2 | 2 | 2 | 1 | 0 | 2 | 0 | 2 | 0 | 0 | 2 | 0 | N/A | N/A | N/A | 1.2 |
|  | 2 | 2 | 2 | 2 | 2 | 2 | 0 | 0 | 2 | N/A | 1 | N/A | 2 | 0 | 2 | 1 | 2 | 2 | 0 | 2 | 0 | 1 | 0 | 0 | 0 | 0 | N/A | N/A | N/A |  |
| Fan, et al [11] | 2 | 2 | 2 | 0 | 2 | 2 | 0 | 0 | 0 | 0 | 0 | 2 | 2 | 0 | 2 | 2 | 2 | 2 | 2 | 2 | 2 | 2 | 0 | 0 | 2 | 0 | N/A | N/A | N/A | 1 |
|  | 2 | 2 | 2 | 1 | 2 | 2 | 0 | 0 | 0 | 0 | 0 | 0 | 2 | 0 | 0 | 2 | 2 | 2 | 0 | 2 | 0 | 2 | 0 | 0 | 2 | 0 | 1 | 0 | 0 |  |

| **Table 2**. Continued | | | | | | | | | | | | | | | | | | | | | | | | | | | | | | |
| --- | --- | --- | --- | --- | --- | --- | --- | --- | --- | --- | --- | --- | --- | --- | --- | --- | --- | --- | --- | --- | --- | --- | --- | --- | --- | --- | --- | --- | --- | --- |
| Study | Introduction | | | Methods | | | | | | | | | | Results | | | | | | | Conflicts | | | Quantitative study | | | Qualitative criteria | | | Quality scores |
|  | Rationale | Objectives | Logic model | Study design | Outcomes | Data collection methods | Participant eligibility | Recruitment | Bias | Sampling | Setting and location | Comparator | Data sources | Enrolment | Description of study population | Reporting on outcomes | Summary of evidence | Limitations | Generalizability | Conclusions | Funding | Ethical considerations | Competing interests | Confounding | Statistical methods | Missing data | Analytical methods | Data validation | Reflexivity of |  |
| Fischinger, et al [25] | 2 | 2 | 2 | 0 | 2 | 2 | 1 | 0 | 0 | 0 | 0 | N/A | 0 | 0 | 0 | 2 | 1 | 2 | 0 | 2 | 2 | 0 | 0 | 0 | 0 | 0 | N/A | N/A | N/A | 1 |
|  | 2 | 2 | 0 | 0 | 2 | 2 | 1 | 0 | 0 | 1 | 2 | N/A | 0 | 0 | 0 | 2 | 2 | 0 | 0 | 2 | 2 | 0 | 0 | 0 | 0 | 0 | N/A | N/A | N/A |  |
| Huisman, et al [26] | 2 | 2 | 1 | 1 | 2 | 2 | 0 | 0 | 1 | N/A | 2 | N/A | 2 | 0 | 2 | 2 | 2 | 2 | 2 | 2 | 2 | 2 | 2 | 0 | 0 | 0 | 2 | 0 | 0 | 1.3 |
|  | 2 | 2 | 2 | 2 | 2 | 2 | 0 | 0 | 1 | N/A | 2 | N/A | 2 | 0 | 1 | 2 | 2 | 2 | 2 | 2 | 2 | 2 | 2 | 0 | 0 | 0 | 2 | 0 | 0 |  |
| Koh, et al [31] | 2 | 2 | 2 | 2 | 2 | 2 | 2 | 2 | 0 | 2 | 1 | 2 | 2 | 2 | 2 | 2 | 2 | 2 | 2 | 2 | 0 | 2 | 0 | 0 | 2 | 0 | N/A | N/A | N/A | 1.5 |
|  | 2 | 2 | 0 | 2 | 2 | 2 | 2 | 2 | 0 | 2 | 2 | 2 | 2 | 2 | 2 | 2 | 2 | 2 | 2 | 2 | 0 | 2 | 0 | 0 | 2 | 0 | N/A | N/A | N/A |  |
| Inoue, et al [27] | 2 | 2 | 2 | 1 | 2 | 1 | 0 | 0 | 1 | 0 | 0 | N/A | 0 | 0 | 2 | 2 | 1 | 2 | 0 | 2 | 2 | 0 | 0 | N/A | N/A | N/A | 1 | 0 | 0 | 1.1 |
|  | 2 | 2 | 2 | 0 | 2 | 2 | 2 | 0 | 0 | 0 | 0 | N/A | 2 | 0 | 2 | 2 | 2 | 2 | 2 | 2 | 0 | 0 | 0 | N/A | N/A | N/A | 1 | 0 | 0 |  |
| Keizer, et al [28] | 2 | 2 | 2 | 1 | 2 | 2 | 2 | 2 | 1 | 0 | 0 | 2 | 2 | 0 | 2 | 2 | 2 | 2 | 2 | 2 | 2 | 2 | 2 | 0 | 2 | 0 | 1 | 0 | 0 | 1.5 |
|  | 2 | 2 | 2 | 0 | 2 | 2 | 2 | 2 | 1 | 0 | 2 | 2 | 2 | 2 | 1 | 2 | 2 | 2 | 2 | 2 | 2 | 2 | 2 | 0 | 2 | 0 | 1 | 0 | 0 |  |
| Khosla, et al [29] | 2 | 2 | 2 | 1 | 2 | 2 | 0 | 2 | 1 | 0 | 2 | N/A | 2 | 0 | 2 | 2 | 2 | 2 | 0 | 2 | 0 | 2 | 0 | 0 | 0 | 0 | 2 | 2 | 0 | 1.2 |
|  | 2 | 2 | 2 | 2 | 2 | 2 | 0 | 2 | 1 | 0 | 2 | N/A | 2 | 0 | 1 | 2 | 2 | 2 | 0 | 2 | 0 | 2 | 0 | 0 | 0 | 0 | 2 | 2 | 0 |  |
| Khosla, et al [30] | 2 | 2 | 2 | 0 | 2 | 2 | 0 | 0 | 0 | 0 | 1 | N/A | 2 | 2 | 2 | 2 | 2 | 0 | 2 | 2 | 0 | 0 | 0 | 0 | 2 | 0 | N/A | N/A | N/A | 1.2 |
|  | 2 | 2 | 2 | 0 | 2 | 2 | 0 | 0 | 0 | 0 | 2 | N/A | 2 | 2 | 2 | 2 | 2 | 0 | 2 | 2 | 0 | 0 | 0 | 0 | 2 | 0 | N/A | N/A | N/A |  |
| Kramer, et al [32] | 2 | 2 | 2 | 1 | 2 | 2 | 2 | 2 | 1 | 0 | 1 | 2 | 0 | 1 | 1 | 2 | 2 | 1 | 2 | 2 | 0 | 0 | 0 | 0 | 2 | 0 | N/A | N/A | N/A | 1.4 |
|  | 2 | 2 | 2 | 2 | 2 | 2 | 2 | 2 | 1 | 0 | 1 | 2 | 0 | 1 | 0 | 2 | 2 | 2 | 2 | 2 | 0 | 2 | 0 | 2 | 2 | 0 | N/A | N/A | N/A |  |
| Liang, et al [33] | 2 | 2 | 2 | 2 | 2 | 2 | 2 | 2 | 1 | 2 | 2 | 2 | 2 | 2 | 2 | 2 | 2 | 2 | 2 | 2 | 2 | 2 | 2 | 0 | 2 | 0 | N/A | N/A | N/A | 1.7 |
|  | 2 | 2 | 0 | 2 | 2 | 2 | 2 | 2 | 0 | 0 | 2 | 2 | 2 | 2 | 2 | 2 | 2 | 2 | 2 | 2 | 2 | 2 | 2 | 0 | 2 | 0 | N/A | N/A | N/A |  |
| Libin, et al [34] | 2 | 2 | 2 | 1 | 2 | 2 | 0 | 2 | 1 | 0 | 0 | 2 | 2 | 0 | 2 | 2 | 2 | 2 | 0 | 0 | 2 | 0 | 0 | 0 | 2 | 0 | N/A | N/A | N/A | 1.4 |
|  | 2 | 2 | 2 | 2 | 2 | 2 | 0 | 2 | 1 | 0 | 1 | 2 | 2 | 2 | 1 | 2 | 2 | 2 | 2 | 2 | 2 | 0 | 0 | 0 | 2 | 0 | N/A | N/A | N/A |  |
| Moyle, et al [35] | 2 | 2 | 2 | 2 | 2 | 2 | 2 | 2 | 1 | 2 | 2 | 2 | 2 | 0 | 2 | 2 | 2 | 2 | 0 | 2 | 2 | 2 | 2 | 0 | 2 | 2 | N/A | N/A | N/A | 1.8 |
|  | 2 | 2 | 0 | 2 | 2 | 2 | 2 | 2 | 0 | 2 | 2 | 2 | 2 | 0 | 2 | 2 | 2 | 2 | 2 | 2 | 2 | 2 | 2 | 2 | 2 | 2 | N/A | N/A | N/A |  |

| **Table 2**. Continued | | | | | | | | | | | | | | | | | | | | | | | | | | | | | | |
| --- | --- | --- | --- | --- | --- | --- | --- | --- | --- | --- | --- | --- | --- | --- | --- | --- | --- | --- | --- | --- | --- | --- | --- | --- | --- | --- | --- | --- | --- | --- |
| Study | Introduction | | | Methods | | | | | | | | | | Results | | | | | | | Conflicts | | | Quantitative study | | | Qualitative criteria | | | Quality score |
|  | Rationale | Objectives | Logic model | Study design | Outcomes | Data collection methods | Participant eligibility | Recruitment | Bias | Sampling | Setting and location | Comparator | Data sources | Enrolment | Description of study population | Reporting on outcomes | Summary of evidence | Limitations | Generalizability | Conclusions | Funding | Ethical considerations | Competing interests | Confounding | Statistical methods | Missing data | Analytical methods | Data validation | Reflexivity of |  |
| Obayashi, et al [36] | 2 | 2 | 2 | 2 | 2 | 2 | 2 | 2 | 2 | 0 | 2 | 2 | 2 | 2 | 2 | 2 | 2 | 2 | 2 | 2 | 2 | 2 | 2 | 0 | 2 | 0 | N/A | N/A | N/A | 1.8 |
|  | 2 | 2 | 2 | 2 | 2 | 2 | 2 | 2 | 2 | 0 | 2 | 2 | 2 | 2 | 2 | 2 | 2 | 2 | 2 | 2 | 2 | 2 | 2 | 0 | 2 | 0 | N/A | N/A | N/A |  |
| Pripfl, et al [37] | 2 | 2 | 0 | 0 | 2 | 1 | 2 | 2 | 1 | 0 | 0 | N/A | 0 | 0 | 2 | 2 | 1 | 2 | 0 | 1 | 2 | 2 | 2 | 0 | 2 | 0 | 2 | 0 | 0 | 1.4 |
|  | 2 | 2 | 0 | 1 | 2 | 2 | 0 | 2 | 1 | 0 | 2 | N/A | 0 | 0 | 1 | 2 | 1 | 2 | 2 | 2 | 2 | 2 | 2 | 2 | 2 | 0 | 2 | 0 | 0 |  |
| Pu, et al [38] | 2 | 2 | 0 | 2 | 2 | 2 | 2 | 2 | 1 | 2 | 1 | N/A | 2 | 0 | 2 | 2 | 2 | 2 | 0 | 2 | 2 | 2 | 2 | N/A | N/A | N/A | 2 | 2 | 0 | 1.8 |
|  | 2 | 2 | 2 | 2 | 2 | 2 | 2 | 2 | 1 | 0 | 2 | N/A | 2 | 0 | 2 | 2 | 2 | 2 | 2 | 2 | 2 | 2 | 2 | N/A | N/A | N/A | 2 | 2 | 0 |  |
| Schussler, et al [39] | 2 | 2 | 2 | 2 | 2 | 2 | 2 | 2 | 1 | 2 | 2 | 2 | 2 | N/A | N/A | N/A | N/A | 2 | 2 | N/A | 2 | 2 | 2 | 0 | 2 | 0 | 2 | 0 | 0 | 1.5 |
|  | 2 | 2 | 0 | 2 | 2 | 2 | 2 | 2 | 1 | 2 | 2 | 2 | 2 | 2 | N/A | N/A | N/A | N/A | N/A | N/A | 2 | 2 | 2 | 0 | 2 | 0 | 2 | 0 | 0 |  |
| Valenti, et al [43] | 2 | 2 | 2 | 2 | 2 | 2 | 2 | 2 | 2 | 0 | 2 | 2 | 2 | 2 | 2 | 2 | 2 | 2 | 2 | 2 | 2 | 2 | 2 | 2 | 2 | 0 | N/A | N/A | N/A | 1.7 |
|  | 2 | 2 | 2 | 2 | 2 | 2 | 2 | 2 | 2 | 0 | 2 | 2 | 2 | 2 | 0 | 2 | 2 | 2 | 2 | 2 | 2 | 2 | 2 | 0 | 2 | 0 | N/A | N/A | N/A |  |
| Sung, et al [40] | 2 | 1 | 2 | 2 | 2 | 2 | 2 | 2 | 1 | 0 | 1 | 2 | 2 | 0 | 2 | 2 | 2 | 2 | 2 | 2 | 0 | 2 | 0 | 0 | 2 | 0 | N/A | N/A | N/A | 1.6 |
|  | 2 | 2 | 2 | 2 | 2 | 2 | 2 | 2 | 1 | 0 | 1 | 2 | 2 | 0 | 2 | 2 | 2 | 2 | 2 | 2 | 2 | 2 | 0 | 2 | 2 | 0 | N/A | N/A | N/A |  |
| Thodberg, et al [41] | 2 | 2 | 2 | 2 | 2 | 2 | 2 | 2 | 1 | 0 | 1 | 2 | 2 | 2 | 2 | 2 | 2 | 2 | 0 | 0 | 2 | 2 | 0 | 0 | 2 | 0 | N/A | N/A | N/A | 1.5 |
|  | 2 | 2 | 0 | 2 | 2 | 2 | 2 | 2 | 1 | 0 | 2 | 2 | 2 | 2 | 2 | 2 | 2 | 2 | 0 | 2 | 2 | 2 | 0 | 0 | 2 | 0 | N/A | N/A | N/A |  |
| Torta, et al [46] | 2 | 2 | 2 | 0 | 2 | 2 | 0 | 2 | 1 | 0 | 2 | N/A | 2 | 0 | 1 | 2 | 2 | 0 | 0 | 2 | 2 | 0 | 0 | 2 | 2 | 0 | 0 | 0 | 0 | 1 |
|  | 2 | 2 | 2 | 2 | 2 | 2 | 0 | 2 | 1 | 0 | 2 | N/A | 2 | 0 | 0 | 2 | 2 | 0 | 0 | 2 | 2 | 0 | 0 | 0 | 2 | 0 | 0 | 0 | 0 |  |
| Wada, et al [44] | 2 | 2 | 2 | 0 | 2 | 2 | 0 | 0 | 1 | 0 | 2 | N/A | 2 | 2 | 2 | 2 | 2 | 1 | 0 | 2 | 0 | 2 | 0 | 0 | 2 | 0 | N/A | N/A | N/A | 1.2 |
|  | 2 | 2 | 2 | 0 | 2 | 2 | 0 | 0 | 0 | 0 | 2 | N/A | 2 | 0 | 2 | 2 | 2 | 2 | 2 | 2 | 0 | 2 | 0 | 0 | 2 | 0 | N/A | N/A | N/A |  |
| Wada, et al [45] | 2 | 2 | 2 | 0 | 2 | 2 | 0 | 0 | 1 | 0 | 2 | 0 | 2 | 0 | 2 | 1 | 1 | 0 | 0 | 2 | 0 | 0 | 0 | 0 | 2 | 0 | N/A | N/A | N/A | 1 |
|  | 2 | 2 | 2 | 0 | 2 | 2 | 0 | 0 | 0 | 0 | 2 | 0 | 2 | 0 | 2 | 2 | 2 | 2 | 2 | 2 | 0 | 0 | 0 | 0 | 1 | 0 | N/A | N/A | N/A |  |
| Zsiga, et al [42] | 2 | 2 | 2 | 0 | 2 | 2 | 2 | 2 | 0 | 0 | 1 | N/A | 2 | 0 | 2 | 2 | 1 | 0 | 0 | 2 | 2 | 2 | 2 | 0 | 1 | 0 | N/A | N/A | N/A | 1.3 |
|  | 2 | 2 | 2 | 0 | 2 | 2 | 2 | 2 | 0 | 0 | 1 | N/A | 1 | 0 | 2 | 2 | 2 | 0 | 2 | 2 | 2 | 2 | 2 | 0 | 1 | 0 | N/A | N/A | N/A |  |
| Note. The highlighted rows indicate the second reviewer’s scores. Total agreement percentage= 86.11 | | | | | | | | | | | | | | | | | | | | | | | | | | | | | | |

The quality of included articles was appraised independently by two authors, through the quality assessment of digital health interventions within the Monitoring and Evaluating Digital Health Interventions framework established by the World Health Organization (WHO) [14].
