# Supplementary material for: Methodologies Used to Study the Feasibility, Usability, Efficacy, and Effectiveness of Social Robots For Elderly Adults: Scoping Review
Source: J Med Internet Res. 2022 Aug 1;24(8):e37434. doi: 10.2196/37434 (PMC9379790; doi:10.2196/37434)
Supplement: Multimedia Appendix 2 [file jmir_v24i8e37434_app2.docx]

| **Table 3.** Characteristics and methodologies of the studies | | | | | |
| --- | --- | --- | --- | --- | --- |
| Author | Schussler, et al. [39] | Pu, et al. [38] | Bajones, et al. [16] | Olde, et al. [28] | Huisman, et al. [26] |
| Robot | PEPPER | PARO | HOBBIT PT2 | NAO | ZORA |
| Aim of Robot | To provide social interaction | To provide companionship | To enable older adults to independently live longer in their own homes | To provide physical exercise and monitoring questionnaire | To provide rehabilitation practice, social activities, and entertainment |
| Aim of study | To explore the effects of PEPPER with a tablet PC–based dementia training program versus a tablet PC–based dementia training program on psychosocial and physical outcomes of PwD, caregivers and dementia trainers | To explore the impact of Paro on managing pain and mood in people with mild to moderate dementia and chronic pain | To explore the acceptability and usability of Hobbit in older adults in their private homes | To uncover the usability and user experience of NAO and monitor and train the health of frail older adults | To monitor and evaluate the feasibility and usability of the care robot Zora is used in daily practice |
| Type of outcome measure | Feasibility, usability, and effectiveness | Efficacy | Feasibility and usability | Feasibility and usability | Feasibility, usability, and effectiveness |
| Study design | Mixed method design | descriptive qualitative approach nested within a pilot randomized controlled trial | Field trial | Mixed methods | Mixed methods |
| Study sample | 40 PWD and their relatives  5 professional caregivers | 11 PwD aged 65 and older | 16 older adults aged 75-89 y. (79.75) | 20 older adults, aged 70 years or older | Older adults with a high intense care demand Care professionals |
| Study setting | Private households | Residential aged care facilities | Private home | An organization for elderly care | 14 care organizations |
| Methodology of data collection | Semi-structured observation Interview Questionnaires and scales | Audio recording  Interview | Questionnaires and scales  Interview | Interview  Questionnaire  Voice and video recordings | Observation  Interview  Questionnaire |
| Interaction Scenario | 3 wk. of home intervention for each person | Individually interacting with PARO for 30 min., 5 d. a week for 6 wk. | 3 wk. of intervention for each participant; freely decide on how and when to interact | 2 modules: monitoring the frailty status and physical exercises for each individual, single session (individual) | Care professionals: choose to use ZORA for any purpose for entertainment and rehabilitation. Clients: Group’s activities included the participation of six to ten clients |
| Relevant outcome measures | Motivation  Care burden QoL Care dependency Mobility Cognitive state Depressive symptoms Affect Behavioral problems  Acceptance and Usability | Mood Pain Attitudes on Paro | Usability  Acceptance | User experience: enjoyment and control  Usability  Acceptance | Facilitators and barriers prior to the use of Zora (usability) Professionals’ views on Zora and the added value for clients  Mood  Involvement |
| Measurement instruments | AES, The Zarit Burden Interview, D-QoL, CDS, TUG, MoCA, GDS, PANAS, NPI, TUI | Interview questions | Interview, NARS, Self-developed items on emotional attachment and perceived reciprocity, FES, Self-efficacy scale, | 5-point Likert scale for enjoyment and control, SUS, Three statements for perceived usefulness, Interpretation of voice and video records | USE, Interview questions, Observation form, Mood scale, Involvement scale |
| Note. AES, The Apathy Evaluation Scale; CDS, The Care Dependency Scale; D-QOL, Dementia Quality of Life; d, day; FES, Falls Efficacy Scale; GDS, Geriatric Depression Scale; h, hour; min, minute; MCI, Mild Cognitive Impairment; mo, month; MoCA, Montreal Cognitive Assessment; NPI, The Neuropsychiatric Inventory; NARS, Negative Attitudes Towards Robots Scale; PwD, People with Dementia; PANAS, The Positive and Negative Affect Schedule; TUG, The Timed UP and GO Test; USE, Usefulness, Satisfaction, Ease of Use; TUI, The Technology Usage Inventory; wk, week; y, year. | | | | | |

| **Table 3**. Continued | | | | | |
| --- | --- | --- | --- | --- | --- |
| Author | Khosla, et al. [29] | Barrett, et al. [18] | Zsiga, et al. [42] | Obayashi, et al. [36] | Koh, et al. [31] |
| Robot name | BETTY | MARIO KOMPAÏ | KOMPAÏ | AI SENSE, SOTA, PALRO | PARO |
| Aim of Robot | To provide social engagement and interaction | To provide companionship and reduce loneliness and social isolation in PwD | To provide cognitive assistance for elderlies to remain autonomous in their homes | To provide social interactions | To improve emotional stability, communication, and motivation in elderly PwD |
| Aim of study | To study the engagement and robot experience of older PwD while interacting with Betty in the context of home-based care. | To evaluate the acceptability and usability and any short-term effect of MARIO on QoL, depression, and perceived social support in PwD | To test a companion robot supporting older adults in their home environments | To investigate the influence of socially assistive robots on activities and social participation of elderlies. | To investigate the effects of PARO on the cognition, emotion, problem behavior, and social interaction of elderly PwD |
| Type of outcome measure | Feasibility, usability, and effectiveness | Feasibility, usability, and efficacy | Feasibility and usability | Effectiveness | Efficacy |
| Study design | Mixed methods | Single group, pre-post, pilot study | Field test | Quasi-experimental design | A nonequivalent control group pretest-posttest design |
| Study sample | 5 PwD aged 75–85 y. | 10 PwD (mean age 83 y., SD 10.1) | 8 seniors, aged 70–83 (77.125 years) | 67 aged 65 y. and over participated (55 women, 10 men, 86.6±8.0) | 33 elderly PwD age 65 y. and older (86.8±6.42) |
| Study setting | Five Australian households | Nursing home | User’s home | Nursing homes | Nursing home facility |
| Methodology of data collection | Video recording  Questionnaire | Questionnaires and scales  Observation | Logfile  Reports  questionnaire | Observation and questionnaire | Questionnaire   Observation |
| Interaction Scenario | Robot installed in every participant’s home for 3 mo. with an average of 22-36 min. daily interactions | Engaged with MARIO BESPOKE applications of their choice, 3 times per week for 4 wk. and 60 min. each participant | Each user received a robot deployed in their home for about 3 mo. | The robot remained installed at the bedside table of each participant for 16 wk.: Morning call and check, recording daily life, drug compliance, evening emergency call | Group intervention twice a week for 6 wk.: Introducing PARO, being friendly and expressing emotions to PARO, recognizing and hugging PARO, remembering, sleeping, feeding, bathing, decorating, separating, and caring for PARO |
| Relevant outcome measures | Engagement  Robot experience | QoL  Depression   Perceived social support | Acceptance  Usability | People's activity and participation | Cognitive function  Emotion  Problem behaviors  Social interaction |
| Measurement instruments | Video coding (duration and frequency of interaction, engagement indicators) (Jones et al., 2015), Robot experience survey (TAM) | QoL-AD, CSDD, MSPSS, Bespoke Questionnaires, Researcher observations questionnaire | CRF, 5-point Likert ratings of robotic functions | Observation sheet, 7-point activity assessment scale | K-CMAI, MMSE-K, AER, Videotape analysis (Observation protocol by Wada et al) |
| Note. AER, Apparent Emotion Rating Instrument; CRF, Case Report Forms; CSDD, Cornell Scale for Depression in Dementia; d, day; h, hour; K-CMAI, Korean version of the Cohen-Mansfield Agitation Inventory; LTCF, Long-Term Care Facility; MSPSS, Multidimensional Scale of Perceived Social Support; min, minute; mo, month; MMSE-K, Korean Mini-Mental State Examination; SD, standard deviation; QoL-AD, Quality of Life- Alzheimer disease; PwD, People with Dementia; TAM, Technology Acceptance Model; wk, week; y, year; | | | | | |

| **Table 3**. Continued | | | | | |
| --- | --- | --- | --- | --- | --- |
| Author | Cavallo, et al. [22] | Liang, et al. [33] | Fan, et al. [11] | Chu, et al. [24] | Beer, et al. [19] |
| Robot name | ROBOT-ERA SYSTEM: ORO, CORO, DORO | PARO | ROCARE/NAO | JACK and SOPHIE | PERSONAL ROBOT2 (PR2) |
| Aim of Robot | To support independent living for older adults | To provide companionship | Interaction with one or more older adults to maintain functional abilities and socialization | To provide emotional and intentional communication and interaction | To provide social interaction and assistance with physical tasks |
| Aim of study | To investigate the acceptance of the Robot-Era system | To investigate the affective, social, behavioral, and physiological effects of PARO for PwD in both a daycare center and a home setting | Presentation of robotic architecture and study of the usability for single and multi-user interaction and engagement of the ROCARE | To show the impact of engagement between two social robots and PwD on QoL in Australian residential care facilities | To study how trialability, demonstrability, or observability influence acceptance of social robots |
| Type of outcome measure | Feasibility and usability | Efficacy | Feasibility and usability | Effectiveness | Feasibility and usability |
| Study design | Cross-sectional | Pilot block randomized controlled trial | Mixed methods | Cross-sectional | Mixed methods |
| Study sample | 45 older persons, aged 65-86 years; 22 women and 13 men, mean age 74.97 (SD 5.70) y.  22 women and 11 men, 73.45 (SD 6.27) y. | 30 PwD aged 67-98 years and their informal caregivers aged 30-86. | 11 older adults, aged 66-94 y. (4 with MCI or dementia); 6 female, 5 male 14 older adults aged 70-90 y. (one pair with MCI/dementia); 9 female, 5 male | 139 PwD (43 males and 96 females, aged 65–90 y.), Care staff | 12 independently living older adults (6 males) aged 68–79 y. |
| Study setting | Laboratory: DomoCasa Lab settings (domestic, condominium, and outdoor) | Two dementia daycare centers and participants’ homes | Laboratory | Residential care facility | Laboratory: Aware Home Research (living room and kitchen area) |
| Methodology of data collection | Questionnaires | Observation  Questionnaire and scales Physiological measures | Questionnaire Video recording Physiological measurement | Scales Observation of video | Questionnaires   Interview |
| Interaction Scenario | Independent task performance, three tasks per participant per session, with a session duration of 3 h. Session 1: the shopping, garbage collection, and communication services Session 2: the reminding, indoor walking support, and outdoor walking support services (individual) | Half an hour of unstructured group sessions with Paro at the daycare center were run 2 to 3 times a week for 6 wk. Participants also had Paro at home for 6 wk. | One-on-one interaction: once, 60 min. Triadic interaction: paired; once, 30 min. | Group-based: 4-6 h., 2 times of every trial for each participant Introduction Song session Game session | Following tasks were demonstrated to the participants in 2.5 h.: (group)  Medication hand-off demonstration  Autonomous learning demonstration  Table clean-up demonstration |
| Relevant outcome measures | Acceptance and Usability | Cognition Depressive symptoms Neuropsychiatric symptoms Behavioral, affective, and social responses Blood pressure and salivary cortisol | Engagement intention, enjoyment of interaction Usefulness  Acceptance | Engagement, care capacity of staff | Acceptance Trialability (perceived usefulness) Demonstrability |
| Measurement instruments | Specific appearance questionnaire, SUS, ad hoc questionnaire on services evaluation | Addenbrooke’s Cognitive Examination, CSDD, Caregiver proxy reports, NPI-Q, CMAI, A rating tool for behavioral tracking | Sensing module (electrophysiological signal collection, gaze estimation, gesture recognition, and speech recognition), RUAS, Pre-post questionnaires on the degree of enjoyment and experiment of provided activities | DCM, WIB, Video review, researcher's note | Robot Opinions Questionnaire, Assistance Preference Checklist, Control methods questionnaire, In-depth interview, Demonstration questionnaire |
| Note. CSDD, Cornell Scale for Depression in Dementia; CMAI, Cohen-Mansfield Agitation Inventory; DMC, Dementia Care Mapping; d, day; h, hour; LTCF, Long-Term Care Facility; min, minute; mo, month; NPI, Neuropsychiatric Inventory; SUS, System Usability Scale; WIB, Well-Being/Ill-Being Scale; wk, week; y, year.   \| **Table 3.** Continued \| \| \| \| \| \| \| --- \| --- \| --- \| --- \| --- \| --- \| \| Author \| Khosla, et al. [30] \| Fischinger, et al. [25] \| Thodberg, et al. [41] \| Pripfl, et al. [37] \| Birks, et al. [20] \| \| Robot name \| MATILDA \| HOBBIT \| PARO \| HOBBIT PT1 and PT2 \| PARO \| \| Aim of Robot \| To deliver diversion therapy services to older PwD \| To enable independent aging in place \| Mental commitment \| The initial goal was to reduce the risk of falling, detect falls and handle emergencies \| To provide cognitive, social, and emotional stimulation \| \| Aim of study \| To study the engagement and acceptability of PwD to Matilda in residential aged care facilities in Australia. \| To evaluate the usability, acceptance, and affordability of Hobbit in elderly adults \| To evaluate quantitative measures of behavior of a nursing home resident, who interacted differently with a real animal/dog compared with interactive and non-interactive fake animals during biweekly animal-assisted visits. \| To evaluate technology market readiness, utility, usability, and affordability of Hobbit under real-world conditions. \| To identify the impact of the use of Paro robot therapy in an aged care facility \| \| Type of outcome measure \| Feasibility and efficacy \| Feasibility and usability \| Effectiveness \| Usability \| Effectiveness \| \| Study design \| Experimental design \| Experimental design \| Randomized complete block design \| Field trial \| Qualitative, descriptive, exploratory \| \| Study sample \| 115 PwD aged 65–90 y. \| 49 participants, aged 70 y. and \| 100 nursing home residents \| 7 older adults age 75 y. and older \| Residents in an aged care facility \| \| Study setting \| Four residential aged care facilities \| Three laboratories in Austria, Sweden, and Greece, a cozy living room \| Nursing home \| Private home \| Aged care facility \| \| Methodology of data collection \| Observation through video record Questionnaire \| Observation Video recording Questionnaire Interview \| Direct observation Video recording Psychiatric scales Interview \| Interview Questionnaires \| Interview \| \| Interaction Scenario \| After the introduction session, group-based interactions were made through singing (multilingual) songs, playing quiz, or storytelling activities, and bingo games. Every trial took 4–6 h. and might repeat more than one time with the same participants. \| Six representative tasks, one trial with a duration of on average 2.5 h. (individual) \| A total of 12 visits for 6 wk., 10 min. each individual. Touching and communicating with dogs, PARO, and the toy cat \| The robot deployed for more than 5 mo., 3 wk. per user \| Daily individual or group therapy sessions for 4 mo., Duration:30-40 mins.  Interaction with Paro includes stroking, cuddling, and speaking to the robotic seal. \| \| Relevant outcome measures \| Emotional, behavioral, verbal, and visual engagement  Acceptability \| Acceptance Usability Affordability \| Behavioral variables including Conversation/Talk, Eye Contact, and Physical Contact Dependency on cognitive function Development in cognitive status, symptoms of depression, and body weight \| Utility Usability Affordability Technology market readiness \| Effectiveness of robot therapy \| \| Measurement instruments \| Video coding based on a Modified version of OERS and Behavioral and visual engagement indicators, Robot acceptability questionnaire (TAM) \| Debriefing questionnaire for acceptance and affordability, Three usability-related questions, SUS \| Video coding (frequency and duration of physical contact), GDS, MMSE, GBS \| Interview, NARS, FES \| Interview \| \| Note. d, day; FES, Falls Efficacy Scale; GDS, Geriatric Depression Scale; GBS, Gottfries-Brane-Steen scale; h, hour; mo, month; MMSE, Mini-Mental State Examination; min, minute; NARS, Negative Attitudes Towards Robots Scale; OERS, Observed Emotion Rating Scale; PwD, People with Dementia; SUS, System Usability Scale; TAM, Technology Acceptance Model; wk, week; y, year. \| \| \| \| \| \| | | | | | |

| **Table 3**. Continued | | | | | | |
| --- | --- | --- | --- | --- | --- | --- |
| Author | Broadbent, et al. [21] | Valenti Soler, et al. [43] | Kramer, et al. [32] | Sung, et al. [40] | Tortat, et al. [46] | Broadbent, et al. [47] |
| Robot name | GUIDE AND CAFERO | PARO AND NAO | AIBO | PARO | NAO | ORBIQ and CAFERO |
| Aim of Robot | To help older adults with basic tasks | Not given | Social stimulation | To provide a social companion | Social agent | To provide services and companionship for elderly adults |
| Aim of study | To investigate any benefits or cause any problems of multiple healthcare robots in an aged care facility. | To compare NAO, PARO, and a real animal (dog) in therapy sessions of PwD in a nursing home and a daycare center | To compare the effects of visitation by a person, a person accompanied by a live dog, and a person accompanied by an AIBO, on behavioral indicators of social interaction among female nursing home residents with dementia. | To evaluate the impact of Paro on the social skills of older adults residing in a residential care facility | To evaluate the short-term and long-term acceptance of NAO in a smart home environment. | To investigate acceptability and feasibility and any impact of social robots on QoL, depression, and medication adherence. |
| Type of outcome measure | Effectiveness and feasibility | Effectiveness | Efficacy | Effectiveness | Feasibility | Feasibility, usability, and efficacy |
| Study design | Non-randomized controlled trial | Randomized controlled block design | Experimental design | Single group pre-posttest design | Experimental design | Repeated measures randomized controlled cross-over trial |
| Study sample | 53 residents  53 staffs | Phase 1: 100 patients aged 58-100 (84.68 years) Phase 2: 110 aged 59-101 (84.7 years) patients (Phase2) PwD | 18 female residents with dementia | 16 residents aged 65 y. or older (77.25 ± 6.7) | 8 participants aged 70-95 (mean 77 y.) in Austria and 8 in Israel | 29 older adults aged 72-94 y. (85.23, SD 5.14) |
| Study setting | Two rest home units and two nursing home units | Nursing home and a daycare center | Home for the aged | Residential care facility | A real-life user apartment in Austria  A Senior Center home in Israel | Private home |
| Methodology of data collection | Questionnaires and scales Interview Observation | Observation of video  Scales | Observation of video | Scales | Questionnaire | Questionnaires and scales Interview |
| Interaction Scenario | Both robots were deployed to residents’ lounges and staff room for approximately 2 mo., from 9 am to 5 pm (individual) | 2 d. per week during 3 mo. with a duration of 30–40 min. each individual Therapeutic activities, including flashcards, practicing the use of everyday objects, sensory stimulation exercises | 3 visits for individuals, 1 visit a week for 3 wk.: the visitor alone, the visitor accompanied by a friendly dog, and the visitor accompanied by AIBO | Interaction with Paro in a group setting, for 30 min., twice a week for 4 wk. | 5 scenarios: asking about the weather conditions, listening to music, physical exercises, receiving an environmental warning, calling a friend, Duration: 3 mo. of long-term trial, 90 min. for each interaction | Robots were installed into the residents’ apartments for 6 wk. |
| Relevant outcome measures | QoL Depression Acceptance | Neuropsychiatric Symptoms Cognitive state  Apathy QoL | Socially interactive behavior | Participation | Acceptance | QoL, depression, and adherence Acceptability and feasibility |
| Measurement instruments | QoL-AD, Proxy QoL ratings, GDS, 3 validated subscales of a dependency scale, AMTS Staffs: SF-12, Job satisfaction measure, PERI, RAS, Mind perception scale, Interview | NPI, GDS, sMMSE, MMSE, APADEM-NH, AI, QUALID, Post-hoc observational analysis | Videotape analysis (Behavior and conversation variables) | ACIS, Activity participation scale | Acceptance questionnaire | SF-12, GDS-15, MARS, Interview, Mind Perception Questionnaire, RAS |
| Note. APADEM-NH, Apathy Scale for Institutionalized PwD Nursing Home version; AI, Apathy Inventory; AMTS, Abbreviated Mental Test Score; ACIS, Assessments of communication and interaction skills; AD, Alzheimer disease; CSDD, Cornell Scale for Depression in Dementia; d, day; GDS, Geriatric Depression Scale; h, hour; QoL, Quality of Life; SF-12, Short Form 12; NPI, Neuropsychiatric Inventory, PwD, People with Dementia; QUALID, Quality of Life in Late-stage Dementia; QoL-AD, Quality of Life- Alzheimer disease; MSPSS, Multidimensional Scale of Perceived Social Support; MARS, Medication Adherence Report Scale; min, minute; mo, month; RAS, Robot Attitudes Scale; SF-12, Short Form 12; sMMSE, Sever Mini-Mental State Examination; wk, week; y, year. | | | | | | |

| **Table 3**. Continued. | | | | | | | |
| --- | --- | --- | --- | --- | --- | --- | --- |
| Author | Cavallo, et al. [23] | Moyle, et al. [35] | Inoue, et al. [27] | Banks, et al. [17] | Wada, et al. [44] | Wada, et al. [45] | Libin, et al. [34] |
| Robot name | ROBOT-ERA SYSTEM: ORO, CORO, DORO | PARO | PAPERO | AIBO | PARO | PARO | NECORO |
| Aim of Robot | To provide practical assistance | To provide companionship | To provide communication | To alleviate loneliness | To provide social interaction | Therapy | To provide emotional communication |
| Aim of study | Evaluation of the technical effectiveness and acceptability of the Robot-Era robotic services | To compare the effect of PARO and interactive reading groups on emotions in people living with moderate to severe dementia in a residential care setting | To evaluate the feasibility and confirm the field-based method through the development of an information support robot PAPERO for PwD | To compare the ability of a living dog (Dog) and a robotic dog (AIBO) to treat loneliness in elderly patients living in LTCF. | To investigate the psychological and social effects of PARO on the residents | To provide an interim report on the effectiveness of PARO in improving the mood of elderly adults | To compare the impact of a robotic cat and a plush toy cat on agitation, affect, and engagement in elderly PwD |
| Type of outcome measure | Feasibility | Efficacy | Feasibility | Effectiveness | Effectiveness | Efficacy | Efficacy |
| Study design | Experimental design | Randomized crossover design | Quasi-experimental | Randomized controlled group design | Quasi-experimental study design | Quasi-experimental study design | Comparison condition experimental design |
| Study sample | 35 persons aged 65-85 y. (mean age 73.8 ± 6.0 y.); cognitively and IADL independent, 13 male, 22 females | 18 residents with mid to late-stage dementia age 65 y. and older | 5 PwD 85.8 ± 7.3 y. | Residents in LTCF | 12 subjects, aged 67–89 years (77.5 ± 7.3) | 14 subjects aged 77-98 (88.2±6.0), mostly with Dementia | 9 cognitively impaired nursing home residents aged 83-98 y. |
| Study setting | Laboratory: the experimental domestic setting and outdoor | Residential care facility | Private home | LTCF | A care house | A health service facility | Nursing home |
| Methodology of data collection | Questionnaire and interviews | Questionnaires and scales | IC recorder | Interview  Scales | Video Recording Urine test | Scale Video recording | Direct observations of behavior |
| Interaction Scenario | Shopping delivery (18 min.) and garbage collection task performance (8 min.), single session (individual) | Both intervention and control activities ran for 45 min., three afternoons per week, for 5 wk. with groups of nine. Activities around the concepts of discovery, engaging an emotional response, social interaction in the group through discussion about PARO, and touching PARO | 5 d. of talking with the robot for about 30 min. each day individually | Weekly group visits lasting 30 min. for 8 wk., in residents’ room | The residents could play with PARO whenever they wished from 8:30 to 18:00 for 2 mo. | 2 d. of interaction per week with a duration of 1 h. for 3 mo.; the robots were placed in the center of a table surrounded by participants | Two interactive sessions for each resident—one with the robotic cat and one with the plush cat. Duration: 10 min. each |
| Relevant outcome measures | Acceptability | QoL Emotions | Acceptability | Loneliness | Social interaction Stress level | Mood | Agitation Affect Engagement |
| Measurement instruments | Self-constructed questionnaire based on UTAUT Model | QoL-AD, Revised Algase Wandering Scale–Nursing Home version, AES, GDS, RAID, OERS | Conversation transcripts analysis (number of positive and negative remarks) | The UCLA Loneliness Scale, MLAPS | Urine tests, Video coding (time spent on interactions) | Face scale | ABMI, Lawton’s Modified Behavior Stream, 5-point scale for engagement level |
| Note. AES, Apathy Evaluation Scale; AMBI, Agitated Behaviors Mapping Instrument, day; h, hour; GDS, Geriatric Depression Scale; LTCF, long-term care facility, min, minute; mo, month; MLAPS, Modified Lexington Attachment to Pets Scale; OERS, Observed Emotion Rating Scale; PwD, People with Dementia; RAID, Rating Anxiety in Dementia Scale; UTAUT, Unified Theory of Acceptance and Use of Technology; wk, week; y, year. | | | | | | | |
