# Supplementary material for: Methodologies Used to Study the Feasibility, Usability, Efficacy, and Effectiveness of Social Robots For Elderly Adults: Scoping Review
Source: J Med Internet Res. 2022 Aug 1;24(8):e37434. doi: 10.2196/37434 (PMC9379790; doi:10.2196/37434)
Supplement: Multimedia Appendix 3 [file jmir_v24i8e37434_app3.docx]

| **Table 4.** Outcomes | | |
| --- | --- | --- |
| Author | Outcome | Study limitations |
| Schussler, et al. [39] | Not available | Results cannot be generalized to other settings and other types of dementia |
| Pu, et al. [38] | Improvements on positive emotion, mood, and relaxation. Some anecdotal evidence of decreased pain in PwD with chronic pain.  PARO was well accepted. Limitations of its weight, voice, and characteristics. | Small sample size Earlier experience with PARO  The short duration of interviews  Variety of data sources The influence of medications on pain and the challenges of pain assessment |
| Bajones, et al. [16] | High-rated usability indicators, the quantitative data did not reveal any increase in user acceptance, the qualitative results do suggest that Hobbit was well received. | Not given |
| Olde Keizer, et al. [28] | Very positive perceived usefulness and enjoyment for both modalities.  Main usability issues for NAO: speech interaction, older adults’ difficulties in human-robot interaction, and a lack of affordances of NAO. | Just one type of social robots Bias in recruiting participants Sample size |
| Huisman, et al. [26] | Several barriers to the use of the robot. Positive influence on clients as an added value for the care professionals in having fun at work. | Not comparable quantitative data  Inconsistent observation method |
| Khosla, et al. [29] | Most of the positive emotion occurs during the singing and dancing activity provided by Betty. Engagement when singing and dancing activity is significantly higher than in other activities, indicating that the participants mostly prefer using the Betty robot for entertainment purposes. | A limited number of participants  Not validated questions adapted from Heerink et al (2010) Not analyzing negative responses of robot |
| Barrett, et al. [18] | No significant differences in QoL, depression, and perceived social support. Might be acceptable. PwD can engage with a social robot in a real-world nursing home. | Small sample size and short duration of intervention Possible measurement bias due to undertaking intervention and observation at the same time by the researcher |
| Zsiga, et al. [42] | The most useful and the least reliable robot functions were navigation and verbal communication. The robot was accepted enthusiastically by the senior subjects. | Not given |
| Obayashi, et al. [36] | Significant improvements in targeted activities and participation, communication, self-care, and social life. | The challenging environment  Heterogeneity in the three types of SARs conversation Technical issues  Small sample size |
| Koh, et al. [31] | Significantly improved positive emotions and social interaction and decrease problem behaviors | Performing intervention program only in the experimental group |
| Cavallo, et al. [22] | Men gave ORO’s appearance an overall score higher than women. Participants younger than 75 years understood more readily the functionalities of Robot-Era robots compared to older people. For the ad hoc questionnaire, the mean overall score was higher than 80 out of 100 points for all Robot-Era services. Older persons with a high educational level gave Robot-Era services a higher score than those with a low level of Education. A higher score was given by male older adults for shopping, indoor walking support, and outdoor walking support. | Not validated appearance questionnaire  2 different samples  Technical problems  Lack of continuity in experimentation format |
| Liang, et al. [33] | Improved facial expressions (affect) and communication with staff (social interaction). Care recipients with less cognitive impairment responded significantly better to Paro. No significant differences in dementia symptoms, nor physiological measures between the intervention and control group. | Difficult recruiting and maintaining participants  Lack of comprehension and inability to provide physiological samples  Small sample size  Not all people consented |
| Fan, et al. [11] | Positive perceptions on acceptability and use of the ROCARE. Improved social communication between pairs of participants in addition to engagement with activities. | Small sample size and short interaction  Offline analysis of electrophysiology signals   Predefined order of the activities |
| Chu, et al. [24] | The findings show that social robots can improve diversion therapy service value to PwD through sensory enrichment, positive social engagement, and entertainment. | Small sample size  5 minutes duration of observation  Only participants with positive attitudes toward the robots |
| Beer, et al. [19] | Positive first impressions on the medication and table cleaning tasks, mixed results about the autonomous learning task. Participants tended to find the robot more useful and were in general more open to accepting the robot after the demonstration, as compared to pre-exposure. This also accounted for a range of specific tasks. | Small sample size Short demonstration period Did not investigate older adults in other settings with other problems  Other age groups may have different attitudes toward robot assistance The robot’s appearance may have influenced user perceptions of it performing a socially-oriented task. The construct ease of use was not often mentioned in the interview. |
| Khosla, et al. [29] | Significant improvements in emotional, visual, and behavioral engagement. Verified acceptance. | Not given |
| Note. PwD, people with dementia; QoL; quality of life. | | |

| **Table 4.** Continued | | |
| --- | --- | --- |
| Author | Outcome | Study limitations |
| Fischinger, et al. [25] | The robot was assessed as usable and acceptable. Participants were skeptical of buying it. | Not given |
| Thodberg, et al. [41] | The dog and the interactive robot seal triggered the most interaction. The higher the cognitive impairment level, the more interaction was directed toward the animal. | Less confident dogs due to not being accompanied by their owners Large dogs |
| Pripfl, et al. [37] | Perceived security did not increase. The utility of the robot’s functions was appreciated. Usability was negatively influenced by a lack of robustness. Not affordable. | Technical issues  Small sample |
| Birks, et al. [20] | Improved emotional state and social interactions. Reduction of challenging behaviors. | Small sample size  single facility |
| Broadbent, et al. [21] | Very mixed responses with positive, neutral, and negative comments. More perceived agency in the intervention group. Decreased perceived agency over time in both groups.  A significant increase in job satisfaction in the control group only. Health-care robots can be acceptable to some people in aged care settings. No significant difference in depression, QoL, and mobility. | Not seeing all the interactions by the staff |
| Valenti Soler, et al. [43] | Improved irritability and global neuropsychiatric symptoms after participating in sessions with the humanoid robot, but not after sessions with the animal-shaped robot. No benefits concerning cognition. Barely shown significant changes in QoL. | Drop out and new inpatients  Randomization  Similar characteristics of the individuals and environment  Some false positives change the high number of comparisons made and small sample sizes |
| Kramer, et al. [32] | Stimulated social interaction in all three types of visits. Both the live dog and AIBO stimulated resident social interaction beyond that stimulated by the visitor alone. The AIBO induced longer looks and more resident-initiated conversation than the live dog. The robotic dog may provide a viable alternative to live animal visitations. | Small sample size with one gender Applicability of the results is limited by the nature and the duration of visits |
| Sung, et al. [40] | Significantly improved communication, interaction skills, and activity participation. | Possibility of the Hawthorne effect Small convenience sample  Extraneous variables such as the impact of contact by staff |
| Torta, et al. [46] | The robot was trusted by the participants. A cross-cultural comparison showed that results were not due to the cultural background of the participants. The perceived enjoyment might decrease over time. | Not given |
| Broadbent, et al. [47] | No significant impact on adherence, depression, or QoL. Significant decrease in the perceived agency, while the robots were feasible and acceptable, improvements in their reliability and functionality may increase their efficacy. | The robots did not cause participants to make mistakes in adherence. |
| Cavallo, et al. [23] | Doro and Coro were considered to be aesthetically pleasing and inspired confidence. Doro was considered too big for a domestic environment by almost half of the participants. The drug and shopping and garbage services were found easy to use. Acceptability of the robots was high. Intention to use was high in case of need. The tendency to use the service was mixed. The success rate for technical task performance of the robots was between 63% and 75%. The service was believed to increase the QoL. | Difficulties in Human-robot interactions through a tablet interface Technical problems with the speech recognition system |
| Moley, et al. [35] | A moderate to large positive influence on participants’ QoL and pleasure in the PARO intervention group compared to the reading group. | Sample size  A large amount of missing data |
| Inoue, et al. [27] | Positive ratings on the acceptability. Suggested the robot to support independent living by persons with dementia. | PwD’s complicated variety of symptoms  Very nascent stage of implementation method |
| Banks, et al. [17] | Aibo group and living dog group had statistically significant. Improvements in their levels of loneliness. No difference between the effectiveness of a living and robotic dog in reducing loneliness. Residents showed high levels of attachment to both the dog and AIBO. | Not given |
| Wada, et al. [44] | Increased social interaction among participants. Significant Improved reactions of the subjects’ vital organs to stress by the Urine test. | Not including a control group Small sample size |
| Wada, et al. [45] | Improved moods and feelings. The robots encouraged elderlies to communicate with each other and with their caregivers. | Limited subject size and experiments |
| Libin, et al. [34] | Not identical, similar trends were seen for the two cats. Significant decrease in agitation and increase in pleasure and interest. No significant differences in engagement parameters. | Small sample size  Gender homogeneity  Short-term sessions |
| Note. PwD, people with dementia; QoL; quality of life. | | |
